# A randomized, double-blind, controlled study on the efficacy of an oral dietary supplement containing fish oil, ASU and phytotherapeutic extracts in canine osteoarthritis

**DOI:** 10.3389/fvets.2025.1693838

**Published:** 2026-02-09

**Authors:** Angela Palumbo Piccionello, Valentina Riccio, Sara Sassaroli, Antonio Tredanari, Felice Ciabocco, Margherita Galosi, Mario Fordellone, Giacomo Rossi, Nicola Pilati, Fabrizio Dini

**Affiliations:** 1 School of Biosciences and Veterinary Medicine, University of Camerino, Matelica, Italy; 2 Department of Mental and Physical Health and Preventive Medicine, University of Campania Luigi Vanvitelli, Naples, Italy

**Keywords:** canine osteoarthritis, gait analysis, multimodal therapy, NSAIDs, nutraceuticals

## Abstract

Osteoarthritis (OA) is a common musculoskeletal disorder in canines, characterized by discomfort, lameness, and reduced mobility. Because of the inconsistent efficacy of available treatments, and that a definitive resolution can be achieved only in a small number of cases, OA remains a major challenge in veterinary orthopedics. The current strategy is usually a multimodal approach involving systemic administration of anti-inflammatory drugs, intra-articular therapies, physiotherapy, dietary modifications, and nutraceuticals. The main goals are to slow progression, preserve joint function, and alleviate pain and inflammation. Although non-steroidal anti-inflammatory drugs (NSAIDs) are effective agents for controlling OA signs, their long-term use is associated with adverse effects. Therefore, in recent years, growing attention has been directed toward nutraceutical compounds. Thanks to their natural origin and safety, these products have shown promising results in reducing pain and inflammation in dogs with spontaneous osteoarthritis, representing a valuable alternative or complementary option. The aim of this study was to evaluate the efficacy of a nutraceutical formulation, mainly composed of fish oil, unsaponificable fraction from Avocado and Soy seeds, Turmeric extract, Devil’s Claw, Boswellia, *Salix alba* extract, *Piper nigrum*, *Haematococcus pluvialis*, Magnesium salt of stearic acid, maltodextrin (Asudyn^®^), by comparing its short- and mid-term clinical, radiographic, and cytological outcomes with those obtained from treatment with mavacoxib (Trocoxil^®^), a selective COX-2 inhibitor. Twenty dogs were enrolled and randomly assigned into two groups. Group A received oral administration of Asudyn^®^ for 90 consecutive days, whereas Group B was treated with Trocoxil^®^ at T0, T15, T45, and T75. Clinical evaluations were conducted at baseline (T0) and at 30, 60, and 180 days after treatment initiation. Lameness was assessed using the Numerical Rating Scale (NRS), pain with the Canine Brief Pain Inventory (CBPI) and Visual Analog Scale (VAS). Additional parameters included limb circumference (CRF), Total Pressure Index (TPI %), and Gait Lameness Score (GLS %). Synovial fluid sampling and radiographic examinations were performed at T0, T30, and T60. The results showed that Asudyn^®^ had efficacy comparable to Trocoxil^®^ in reducing pain, improving lameness, and enhancing synovial fluid quality. Overall, Asudyn^®^ proved as effective as, and sometimes superior to, mavacoxib in managing canine OA.

## 1 Introduction

Osteoarthritis (OA) is a chronic, progressive joint disorder characterized by degeneration of articular cartilage, formation of periarticular osteophytes, and inflammation of the synovial membrane, ultimately resulting in pain and gradual loss of joint function (1, 2). To date, there are not available therapies that can reverse the irreversible progression of osteoarthritis (OA) (3, 4). Consequently, the current standard of care relies on a multimodal approach tailored to the severity of the disease. This strategy aims to alleviate joint pain, reduce disease progression, and improve overall patient quality of life (5–7). The multimodal therapy includes physical therapy, weight management, intra-articular treatments, pharmacological therapies with analgesic and anti-inflammatory agents, and, in some cases, surgical intervention. Among the most commonly used pharmacological treatments for OA are non-steroidal anti-inflammatory drugs (NSAIDs), particularly the selective cyclooxygenase-2 (COX-2) inhibitors. However, the prolonged use of these agents is associated with numerous adverse effects (8, 9). Therefore, due to the high incidence of adverse events related to NSAIDs, natural compounds, known as nutraceuticals have been developed in recent years to provide beneficial pain relief and anti-inflammatory properties without the adverse effects of NSAIDs. Those nutraceuticals are administered through dietary supplements or complementary feeds, often in pill, capsule, or powder form, that are intended to supplement one’s diet with additional nutrients like vitamins, minerals, or herbs (10). In recent decades, product like complementary feeds have gained widespread popularity in veterinary medicine. Many veterinarians now recommend their use in the management of joint diseases, particularly degenerative joint disorders such as OA (11–13). These compounds, including macronutrients, proteins, amino acids, omega-3 fatty acids, and vitamins, appear to not only serve as building blocks for biological processes but also have the potential to support and modulate joint structure and function. Therefore, they are considered by many veterinarians to be a safe and promising alternative (14, 15). Due to their high safety profile and low risk of adverse effects, they are also considered a valuable adjuvant in multimodal treatment protocols, especially when used in combination with pharmacological agents of proven efficacy, depending on the animal’s clinical condition (16). Literature reports several studies on the efficacy of certain natural compounds in reducing primarily inflammation and pain. Among the most studied are the Essential polyunsaturated fatty acids (PUFAs). These products are classified as omega-3, omega-6, or omega-9 depending on the position of the last double bond along the fatty acid chain.

The main dietary PUFAs are omega-3 [such as linolenic acid (ALA), docosahexaenoic acid (DHA), and eicosapentaenoic acid (EPA)] and omega-6 (such as linoleic acidand arachidonic acid). Their main sources, where they are found in high concentrations, are oils extracted from marine animals such as fish (FO), krill (KO), and New Zealand green-lipped mussels (GLM) (17, 18), from plants represented by flaxseed, walnuts, safflower, corn, soybeans, and sunflower seeds, as well as in meat fat. Avocado and soybean unsaponifiables (ASU) are plant extracts derived from unsaponifiable residues of avocado and soybean oils, commonly mixed in a ratio of one-third to two-thirds, respectively (19–22). ASU contain many compounds, including fat-soluble vitamins, sterols, triterpene alcohols, and possibly furanic fatty acids. The main components of ASU include the phytosterols β-sitosterol, campesterol, and stigmasterol (23). The ASU exerts chondroprotective effects and it stimulate collagen synthesis by chondrocytes (22, 24). Devil’s claw is the collective name for plants of the genus Harpagophytum (Pedaliaceae) (25, 26). The secondary root tubers of devil’s claw are used in pharmaceuticals and botanical supplements and are exported from southern Africa, primarily Namibia. The large body of experimental evidence in the scientific literature from 1963 (27) to the present day (28) makes devil’s claw one of the most studied plant extracts, dating back to 1901, when the beneficial properties of this tuber in wound healing were first observed (29). The botanical biochemical composition of Harpagophytum includes iridoid glycosides, primarily harpagoside, harpagide, and procumbide; phytosterols, the parent compounds of which are phenylpropanoids; triterpenes; 3β-acetyloleanolic acid; flavonoids; and unsaturated fatty acids such as cinnamomic acid, chlorogenic acid, and stachyose, identified as the most important compounds present in the root (30). Pharmaceutical studies on devil’s claw have mainly investigated the anti-inflammatory activities caused by some active principles present in the tuber. *Boswellia serrata* is a branched tree native to the hilly regions of India belonging to the Burseraceae family whose aromatic gum resin is widely used for its anti-inflammatory and anti-arthritic properties mainly attributed to the action of boswellic acids (31–34). The resinous part of *Boswellia serrata* contains monoterpenes, diterpenes, triterpenes, tetracyclic triterpenic acids, and four major pentacyclic triterpenic acids: β-boswellic acid, acetyl-β-boswellic acid (AβBA), 11-keto-β-boswellic acid, and acetyl-11-keto-β-boswellic acid (AKBA) (35). Boswellic acids with the characteristic pentacyclic triterpene ring may exhibit actions related to the inflammatory cascade, with AKBA being the most active, selectively inhibiting a branch of the arachidonic acid (5-lipoxygenase) cascade linked to leukotriene production without affecting other LOX and COX activities. Curcuminoids are natural polyphenols found as major components in turmeric, a yellow spice derived from the rhizome of the *Curcuma longa* plant, whose use has long been mentioned in traditional Chinese and Ayurvedic medicin. It contains several bioactive compounds, mainly curcumin, demethoxycurcumin, bis-demethoxycurcumin, and turmeric essential oils, with antioxidant and anti-inflammatory properties due to their ability to modulate several important molecular targets (36). Despite its notable beneficial properties, one of the main critical aspects of the use of curcumin concerns the low bioavailability and rapid metabolism that occur following oral administration (37). Formulations for animal use with high bioavailability have been patented, thus managing to best express the activities offered by this nutraceutical on joint disease (38, 39). There are numerous other natural active ingredients for which studies demonstrate their usefulness in the treatment of joint diseases, among which we remember black pepper, white bark (*Salix alba*), pineapple, algae and green-lipped mussels (40, 41) also in cats (42, 43). A concern with natural compounds is that they are extracted from herbal products or other environmental sources, whose active compound content is influenced by extraction and purification strategies, local plant growing conditions (e.g., soil composition), and the frequently used oral administration route, which is associated with uneven uptake and often inconsistent bioavailability (44). Therefore, the same active ingredients may be more or less effective, depending on multiple factors related to the place of cultivation and the type of extraction, as well as the quantity contained within the commercial product. Despite numerous studies, effective therapeutic concentrations for individual compounds have not yet been definitively established, nor have specific combinations at defined doses that could maximize their *in vivo* efficacy been identified (45–47). Furthermore, some studies have methodological limitations, such as the lack of control groups or the use of inadequate assessment methods to definitively determine the efficacy of these compounds (48, 49). In recent years, a complementary feed (Asudyn^®^) (50), has been available in Italy. It boasts anti-inflammatory and pain-relieving properties. It contains Dicalcium phosphate, hydrolyzed animal proteins, fish oils, extracts from avocado, soy, pineapple, algae, magnesium, maltodextrin, corn starch vitamins E and C, manganese, devil’s claw, boswellia, curcuma, black pepper, white Salix, The purpose of this study is to evaluate the efficacy of this specific nutraceutical formulation (Asudyn^®^) in the treatment of spontaneous osteoarthritis (OA) in dogs, compared to treatment with mavacoxib, a selective cyclooxygenase-2 (COX-2) inhibitor (51, 52).

## 2 Materials and methods

### 2.1 Eligibility criteria and dogs’ enrollment

Twenty client-owned dogs diagnosed with osteoarthritis (OA) and referred to the Veterinary Teaching Hospital of the University of Camerino were enrolled in this study. Informed consent was obtained from all owners prior to participation. Inclusion criteria required that dogs be between 1 and 15 years of age, with no restrictions regarding sex or body weight, and classified as ASA physical status I or II. Eligible subjects had to be free from comorbidities, not pregnant or lactating, and exhibit clinical signs of lameness attributable to OA localized in a single joint. Furthermore, only dogs with radiographically confirmed OA graded between 1 and 4 on the modified Kellgren–Lawrence scale were included. Dogs must not have received anti-inflammatory medications or nutraceutical supplements within 15 days prior to study enrollment. All participating dogs were subjected to a standardized management and feeding protocol, which included restriction from uncontrolled physical activities, allowing only leash walking, and were fed a measured amount of commercial diet to minimize body weight fluctuations throughout the study period (53, 54). Additionally, owners were instructed not to administer any nutraceuticals or anti-inflammatory agents during the entire duration of the study. Subjects requiring any treatments different by those specified for their assigned experimental group were excluded from the trial. The supplement (Asudyn^®^) contained the following ingredients per tablet. Composition: Dicalcium phosphate, hydrolyzed animal proteins, fish oil (5.2%), vegetable oils and fats [unsaponifiable fraction (phytosterols) from avocado (*Persea gratissima*) fruit, unsaponifiable fraction (phytosterols) from soybean (*Glycine max*) seed, soy protein concentrate (from seeds), pineapple (*Ananas comosus* L.) extract, dried seaweed (*Haematococcus pluvialis*)] 0.83%, magnesium stearic acid salt, maltodextrin, corn starch. Nutritional additives: Vitamins: 3a700 Vitamin E 58333.3 mg/kg, 3a300 Vitamin C 8333.3 mg/kg Amino acids: 3c301 D, L-Methionine, technically pure 6666.6 mg/kg Trace elements: 3b506 Manganese chelate of glycine, hydrated 416.6 mg/kg. Sensory additives: Botanically defined natural products: Turmeric (*Curcuma longa* L.) extract 83333.3 mg/kg, Devil’s claw (Harpagohytum procumbens DC) extract 18,750 mg/kg, Boswellia (*Boswellia serrata* Roxb. Ex Colebr.) extract 18,750 mg/kg, White willow (*Salix alba* L.) extract 7,500 mg/kg, Black pepper (*Piper nigrum* L.) extract 2,500 mg/kg. Technological additives: E460 microcrystalline cellulose, E551b colloidal silica, 1c322 lecithins (soy lecithin), 1b306(i) tocopherols extracted from vegetable oils (Table 1). Analytical Constituents: Crude protein 4.8; Crude fiber 32.1; Crude oils and fats 14.3; Crude ash 17.0; Moisture 3.9; Calcium 4.75; Magnesium 0.129; Phosphorus 3.47; Methionine 0.84; Manganese 0.0132.

**Table 1:** Table showing the concentration of major components in the phytotherapeutic agent Asudyn^®^.

Composition	Average content per tablet (1,200 mg)
Turmeric extract (*Curcuma longa*)	100 mg
Devil’s Claw dry extract, standardized to 2.5% in harpagoside (from *Harpagophytum procumbens DC*)	22.5 mg
Vitamin C (L-ascorbic Acid)	10 mg
Dried powdered algae, standardized to 2.5% astaxanthin from *Haematococcus pluvialis*	10 mg 2.5 mg
White Willow dry extract, standardized to 25% salicin from *Salix alba*	9 mg
Black Pepper dry extract, standardized to 95% piperine from *Piper nigrum*	3 mg
Vitamin E (all-rac-alpha-tocopheryl acetate)	70 mg
D, L-methionine	8 mg
Manganese Glycinate Chelate, hydrate	500 mcg
Total fish oil concentration	62.4 mg
Total content of Omega-3 fatty acids derived from: Avocado *Persea* unsaponifiable fraction *Glycine max* (Soy) unsaponifiable fraction	40.56 mg
Containing eicosapentaenoic acid (EPA)	26.52 mg
Containing docosahexaenoic acid (DHA)	14.04 mg

### 2.2 Sample size calculation

The sample size was determined *a priori* through simulation-based power analysis using the same linear mixed model specified for the primary analysis, which included treatment group, time points, and the group-by-time interaction as fixed effects, and a random intercept for each subject. Under the assumption of four scheduled assessments and a clinically meaningful between-group difference of moderate magnitude at the final time point, 1,000 simulated datasets indicated that enrolling 10 dogs per group would provide approximately 80% power to detect the prespecified treatment effect at a two-sided significance level of α = 0.05.

### 2.3 Clinical trial procedures

The patients were randomly assigned to two groups, each consisting of 10 dogs. Group A received oral administration of Asudyn^®^ for 90 consecutive days, according to the manufacturer’s leaflet, starting from the day of enrollment (T0), whereas Group B received oral administration of Trocoxil^®^ (Mavacoxib at T0, 15 days after the first administration, and subsequently at 30 and 60 days following the second administration, following the recommended scheme by the manufacturer). At T0 anamnestic data were collected, and the owners’ perception of their dogs’ pain was assessed using the Canine Brief Pain Inventory (CBPI). A thorough general and orthopedic clinical examination was then performed by a specialized clinician, and all findings were recorded in a standardized clinical chart. During the examination, the degree of lameness was assessed through visual gait evaluation, and the circumference (CRF) of the affected limb was measured using a flexible tape measure. Gait analysis was subsequently performed to objectively evaluate locomotor function. Following the clinical assessment, under anesthesia a radiographic study was conducted to confirm the diagnosis and classify the severity of osteoarthritis (OA). Subsequently the joint was aseptically prepared, and synovial fluid was collected via arthrocentesis for qualitative cytological analysis. All sedated procedures were carried out following a standardized anesthetic protocol, as detailed in the corresponding section. For both treatment groups, the clinical examination and pressure platform gait analysis were repeated during follow-up evaluations at 30 days (T1), 60 days (T2), and 180 days (T3) from the initial assessment. Synovial fluid collection and radiographic examination were performed at T0, T1, and T2. At all study time points (T0, T1, T2, T3), the owners were asked to complete the CBPI to assess perceived pain and mobility limitations. All the clinical, radiographic evaluations were conducted by two different expert clinicians, and synovial fluid tests from a pathologist, all of them were in a blinded way.

### 2.4 Anesthesiological protocol

The patients were premedicated with 3 μg/kg of dexmedetomidine and 0.2 mg/kg of methadone administered intramuscularly (IM), followed by induction of general anesthesia with 2–3 mg/kg of intravenous (IV) propofol until tracheal intubation was achieved. Anesthesia was maintained with 1.2% isoflurane in oxygen throughout the procedure.

### 2.5 Canine brief pain inventory

The Canine Brief Pain Inventory (CBPI) (55) enables owners to assess the pain severity perceived in their dogs and the impact of this pain on the dogs’ quality of life. This survey comprises four items concerning the intensity of the dog’s pain and six items detailing how this pain disrupts the dog’s daily routines, totaling 10 items for the owner to evaluate. Each pain-related CBPI item is accompanied by numerical rating scales ranging from 0 to 10, where 0 indicates the absence of pain, and 10 signifies severe pain. Similarly, for the interference items, a score of 0 indicates no interference, while a score of “10″ indicates complete interference. In this clinical investigation, the CBPI was employed as an outcome measure, administered and completed by the owner at T0, T1, T2, and T3 during the trial.

### 2.6 Clinical examination

The head of orthopedic surgery of the University of Camerino conducted this segment of the clinical evaluation, which involved the completion of a standardized clinical form based on the Vesseur-modified lameness classification system (56). This form included several parameters assessed using a numerical scale ranging from 1 (absence of clinical signs of lameness) to 5 (non-weight-bearing lameness). The assessment categories comprised lameness during ambulation, lameness at rest, pain on palpation and evaluation of the contralateral limb. In addition, for each subject, the clinician evaluated pain perception during an initial examination using the Visual Analog Scale (VAS) (57), which employs a numerical severity range from 0 to 10, consistent with the Canine Brief Pain Inventory (CBPI). The clinical assessment further included the measurement of limb circumference using a flexible tape measure, and the Body Condition Score (BCS) of each dog. This clinical evaluation served as a primary clinical outcome measure and was conducted by the same orthopedic specialist at baseline (T0, prior to treatment) and at subsequent follow-up timepoints (T1, T2, and T3) following treatment for each experimental group.

### 2.7 Gait analysis

Pressure sensitive walkway evaluation study was performed using the GAITRite system, which consisted of a pressure-sensitive walkway (GAIT4 Dog. walkway, CIR Systems Inc., Sparta, NJ, United States) and a dedicated software product developed for quadrupeds (GAITFour software version 4.9Wr, CIR Systems Inc., Sparta, NJ, United States). The pressure-sensitive walkway (PSW) utilized in this study comprises encapsulated sensor pads, each with an active surface area of 24 square inches (approximately 61 cm^2^). Each pad integrates 2,304 individual sensors configured in a 48 × 48 matrix, with sensors spaced at 0.5-inch (1.27 cm) intervals. Multiple pads were linked to achieve the necessary walkway length. To demarcate the initiation and termination of each trial, an inactive mat segment measuring 1.25 × 0.85 meters was positioned at both ends of the system. Video data were simultaneously acquired for each trial using digital camera (Logitech C930e HD 1080p) positioned 50 cm above ground level on one side of the PSW. The video recordings were automatically synchronized with the corresponding patient data files. Following data acquisition, a single investigator reviewed all video footage to confirm adherence to predefined inclusion criteria. Subsequent to data processing, a comprehensive report was generated for each subject. For the purposes of this study, pressure-related parameters, specifically the GAIT4Dog^®^ Lameness Score and the Total Pressure Index percentage, were extracted and analyzed for the limb affected by osteoarthritis (OA). Gait assessments were conducted at baseline (T0), and at 30 (T1), 60 (T2), and 180 (T3) days post-treatment. Each dog was guided along the walkway by the same trained handler to ensure consistency. Trials were conducted in a quiet, isolated environment to minimize external stimuli and distractions that could potentially alter the gait pattern. During each trial, dogs were required to traverse the center of the walkway with a loose leash and maintain a forward head position. Walks were repeated multiple times in both directions. A minimum of three comparable trials, characterized by a consistent gait pattern and walking speed, were collected per subject. Each valid trial included at least three complete gait cycles. For inclusion in the final analysis, gait sequences were required to demonstrate regularity and a velocity variability of less than 10% (58, 59). The parameters evaluated in this study included the GAIT4Dog^®^ Lameness Score (GLS) and the Total Pressure Index percentage (TPI%). The GAIT4Dog^®^ Lameness Score (GLS) is a quantitative metric designed to detect reductions in limb loading, which serve as indicators of pain during the stance phase of gait. This score reflects both the degree of unloading (indicative of lameness) and compensatory overloading on the contralateral or adjacent limbs. A GLS value of 100 denotes normal loading and the absence of lameness. Scores below100 represent varying degrees of load reduction on the evaluated limb, with lower scores indicating a greater extent of lameness. The Total Pressure Index percentage (TPI%) represents the proportional weight distribution across all four limbs during locomotion. In a healthy canine gait, this distribution typically approximates 30% for each forelimb and 20% for each hind limb, summing to a total of 100%. Deviations from these values may indicate altered biomechanics due to pain, compensation, or structural abnormalities.

### 2.8 Radiographic examination

Radiographic assessment was conducted to confirm the diagnosis of osteoarthritis (OA) and to obtain orthogonal projections of the joint affected by the degenerative process. Image interpretation was performed by the Head of the Radiology Unit at the Veterinary Teaching Hospital, University of Camerino. Each radiographic examination was evaluated in a blinded fashion, and the severity of OA was graded using a modified version of the Kellgren–Lawrence scale (60) (Table 2). This classification system was based on the evaluation of multiple radiographic features, including the presence of osteophytes, subchondral bone sclerosis, joint space narrowing and/or incongruity, and capsular distension. A numerical grading scale ranging from 0 (no radiographic evidence of OA) to 4 (advanced OA) was used to quantify disease severity, with scores derived from the cumulative assessment of these radiological findings. The modified Kellgren–Lawrence grading was applied at the baseline evaluation (T0) to determine the initial OA status of each subject and was subsequently repeated at 30 days (T1) and 60 days (T2) following treatment, in order to monitor radiographic progression or improvement of the condition over time.

**Table 2:** Radiographic modified scale of osteoarthritis.

Radiographic sign	0	1	2	3	4
Osteophytes	Absence	<1 mm	1–2 mm	2–3 mm	<3 mm
Bone scleroris	Absence	Localized	Pervasive		
Joint narrowing and/or incongruence	Absence	Mild <25%	Moderate 25–50%	Serious >50%	Joint deformity
Capsular ectasia	Absence	Evident			
Final score	0	1–3	4–6	7–9	>10
OA grade	0	1	2	3	4

### 2.9 Synovial fluid examination

Synovial fluid was collected at T0, T1 and T2 via arthrocentesis. A cytological slide was prepared from the collected synovial fluid and subsequently analyzed at the Laboratory of Pathological Anatomy, School of Biosciences and Veterinary Medicine, University of Camerino. Each slide was evaluated and assigned a score from 1 to 4 for the following parameters: synovial lining, polymorphonuclear leukocytes (PMNs), macrophages, lymphocytes, plasma cells, synovial cells, and the presence of red blood cells (RBCs). For each parameter, the score was assigned according to the criteria shown in the Table 3. The total score obtained from the sum of the individual parameter scores was used to determine a severity score. The same procedure was performed for each cytological analysis at every study time point, and the resulting severity scores were then compared and subjected to statistical analysis (61).

**Table 3:** Cytological score for synovial fluid evaluation.

Parameters	Score	Synovial fluid evaluation
Sinovial fluid	1	Synovial fluid in paraphysiological appearance, but slightly diluted (less thick, but continuous smear appearance)
	2	More abundant and diluted synovial fluid. Presence of (a) hemodilution or; (b) serous dilution. Strong reduction in background microgranulometry and poor polymerization of hyaluronic acid. Discontinuous, but thicker smear veil
	3	Very abundant synovial fluid with a strong increase in background protein (exudate). Presence of (a) exudate and hemodilution or; (b) volumetric increase due to protein exudation. Total disappearance of the normal microgranulometry and loss of hyaluronic acid. Highly stainable and continuous smear background
	4	Very abundant and highly proteinaceous synovial fluid (exudate) in the background. Presence of (a) exudate with hemodilution or; (b) volumetric increase due to protein exudation. Total disappearance of the normal microgranulometry and hyaluronic acid. Highly stainable and continuous background smear, with presence of fibrin strands and amorphous fragments/debris
PMNs	1	From 5 to 20
	2	From 20 to 50
	3	From 50 to 150
	4	>150
Macrophages	1	From 2 to 5
	2	From 5 to 20
	3	From 20 to 50
	4	>50
Lymphocytes	1	From 5 to 10
	2	From 10 to 30
	3	From 30 to 80
	4	>80
Plasma cells	1	From 2 to 5
	2	From 5 to 10
	3	From 10 to 30
	4	>30
Synovial cells	1	Some isolated, and sporadic cells (10–15)
	2	Some *micro-clusters* of cells in hypertrophic appearance
	3	Some clusters of cells in hyperplastic appearance
	4	Many clusters of cells in hyperplastic appearance
Red blood cells (erythrocytes)	1	From 50 to 100
	2	From 100 to 200
	3	From 200 to 400
	4	>400

### 2.10 Statistical analysis

Continuous variables were expressed as means and standard deviations or as medians with interquartile ranges (IQRs), depending on their distribution, which was evaluated using the Shapiro–Wilk test for normality. Categorical variables were summarized as absolute frequencies and corresponding percentages. To assess the longitudinal effects of covariates on the continuous outcome variable, Linear Mixed Models (LMMs) were employed. This modeling approach utilizes all available data and appropriately accounts for the correlation among repeated measurements. The covariates included in the model were treatment group (Asudyn^®^, i.e., group A; and Trocoxil^®^, i.e., group B), time point (as a categorical variable), and the interaction between treatment group and time point. Group effects were assessed using Tukey’s *post-hoc* test. For multiple comparisons within the same time point across treatment groups, an ANOVA was conducted, followed by pairwise *t*-tests with Holm-adjusted *p*-values. Similarly, for multiple comparisons within each treatment group across different time points, repeated-measures ANOVA was performed, followed by pairwise *t*-tests with Holm correction for multiple testing. Statistical significance was defined as a *p*-value less than or equal to 0.05. All analyses were performed using R statistical software (version 4.1.3; released on 10 March 2022).

## 3 Results

### 3.1 Enrolled dogs

A total of 20 canine subjects affected by osteoarthritis (OA) and meeting the defined inclusion criteria were enrolled in the study. The subjects were randomly assigned to two groups: Group A, treated with Asudyn^®^, and Group B, treated with Trocoxil^®^ (Mavacoxib). Among the dogs allocated to Group A, there were 4 spayed females and 6 intact males. In Group B, the distribution included 4 spayed females, 3 intact males, and 3 neutered males. No restrictions were applied regarding sex, breed, or affected joint. The age of the subjects ranged from 5 to 12 years, with a mean age of 8.9 years for Group A and 8.4 years for Group B. Group A consisted of 2 German Shepherds, 3 Labradors, and 5 mixed-breed dogs. Group B included 2 Llewellin Setters, 4 Labradors, 2 Great Danes, and 2 mixed-breed dogs. The mean body weight in Group A was 26.48 kg with an average Body Condition Score (BCS) of 5.7, while in Group B the mean body weight was 34.8 kg with an average BCS of 5.5. The affected joints in Group A were: 2 left hips and 1 right hip, 2 left elbows and 1 right elbow, 1 right shoulder and 1 left shoulder, 1 right tarsus and 1 left tarsus. In Group B, the affected joints were: 1 right elbow and 1 left elbow, 3 right tarsi and 1 left tarsus, 1 left carpus, 1 right shoulder and 1 left shoulder.

### 3.2 Clinical evaluation

**Figure 1:**
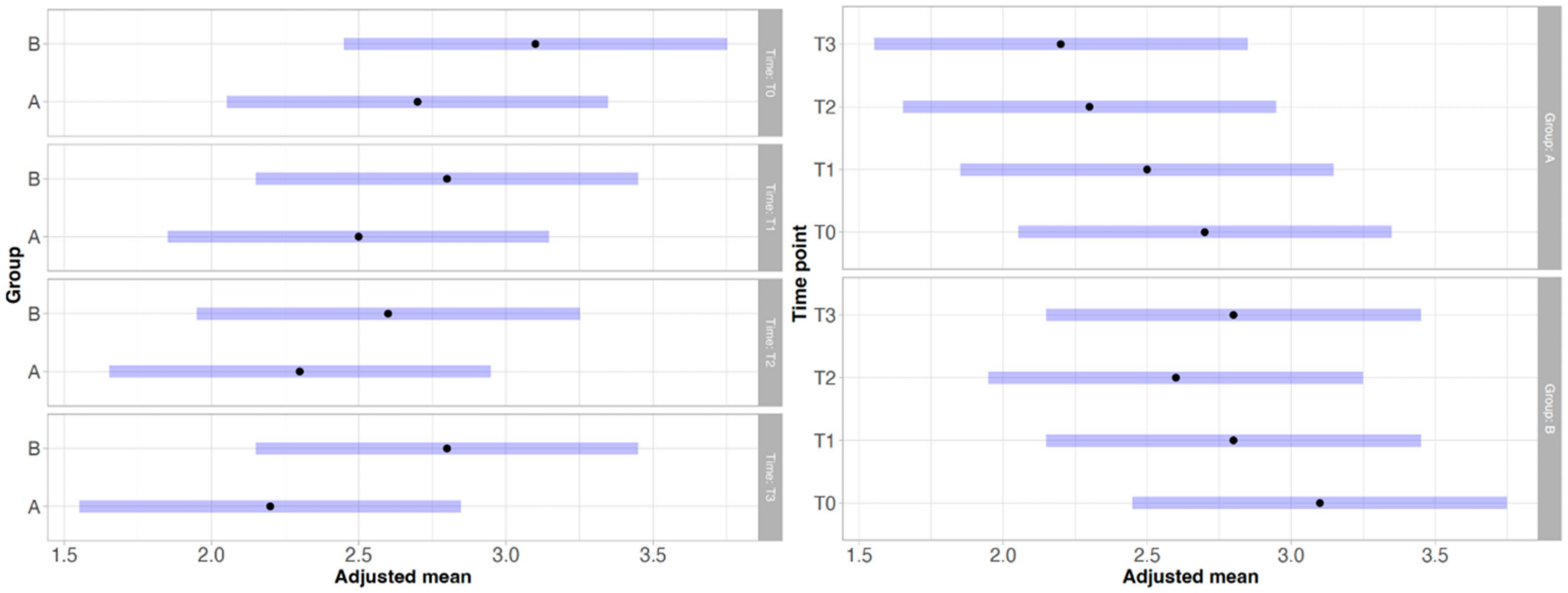
These graphs illustrate the differences for lameness in statistical analysis between the Asudyn^®^ Group (A) and the Trocoxil^®^ Group (B) at the various study time points (left graph), as well as the differences in statistical analysis across study time points within each group (right graph). As shown in the graphs, no statistically significant differences were observed in any of the comparisons performed, as all *p* values were greater than 0.05.

**Figure 2:**
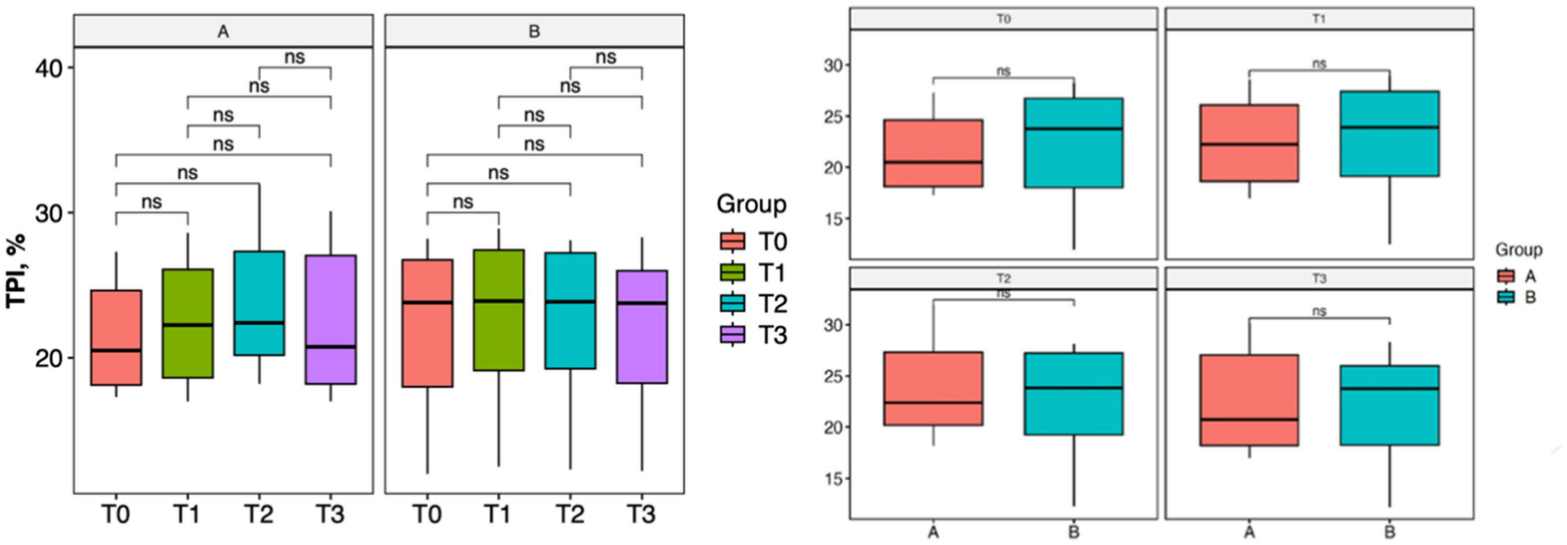
Statistical analysis of the TPI% parameter from gait analysis. Left: intra-group comparisons over time [Asudyn^®^ (A), Trocoxil^®^ (B)]; right: inter-group comparison. No statistically significant differences observed (all *p* values > 0.05).

**Figure 3:**
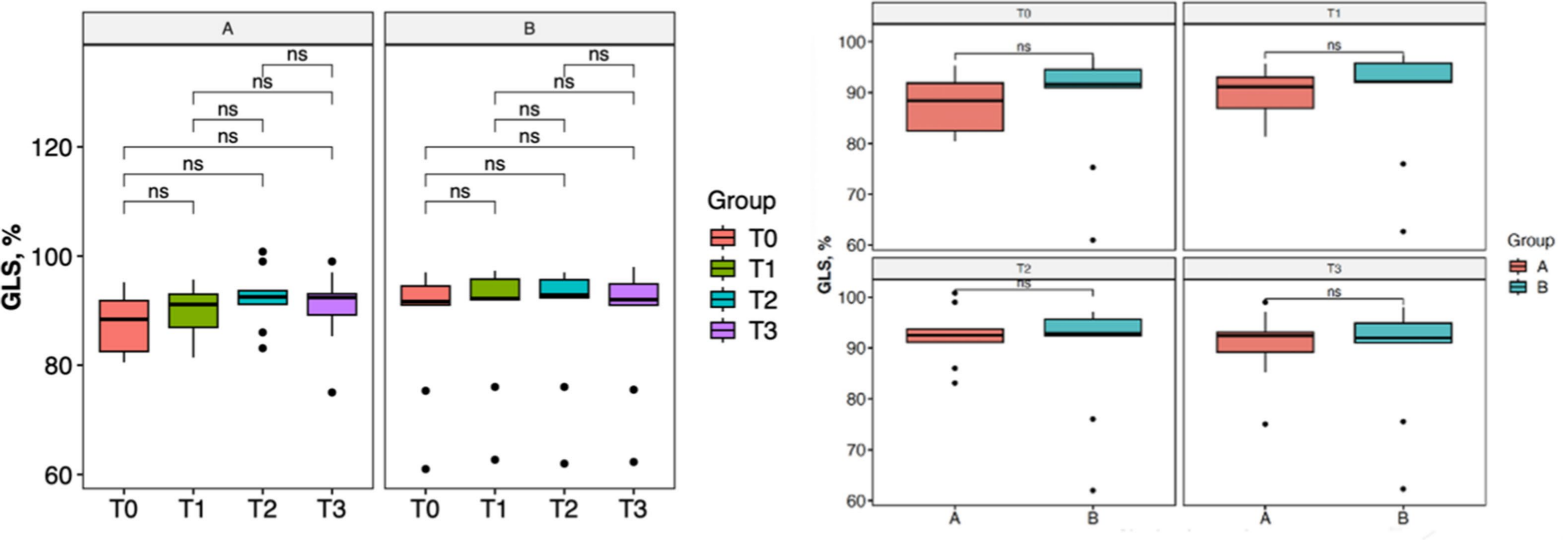
Statistical analysis of the GLS% parameter from gait analysis. Left: intra-group comparisons over time [Asudyn^®^ (A), Trocoxil^®^ (B)]; right: inter-group comparison. No statistically significant differences observed (all *p* values > 0.05).

**Figure 4:**
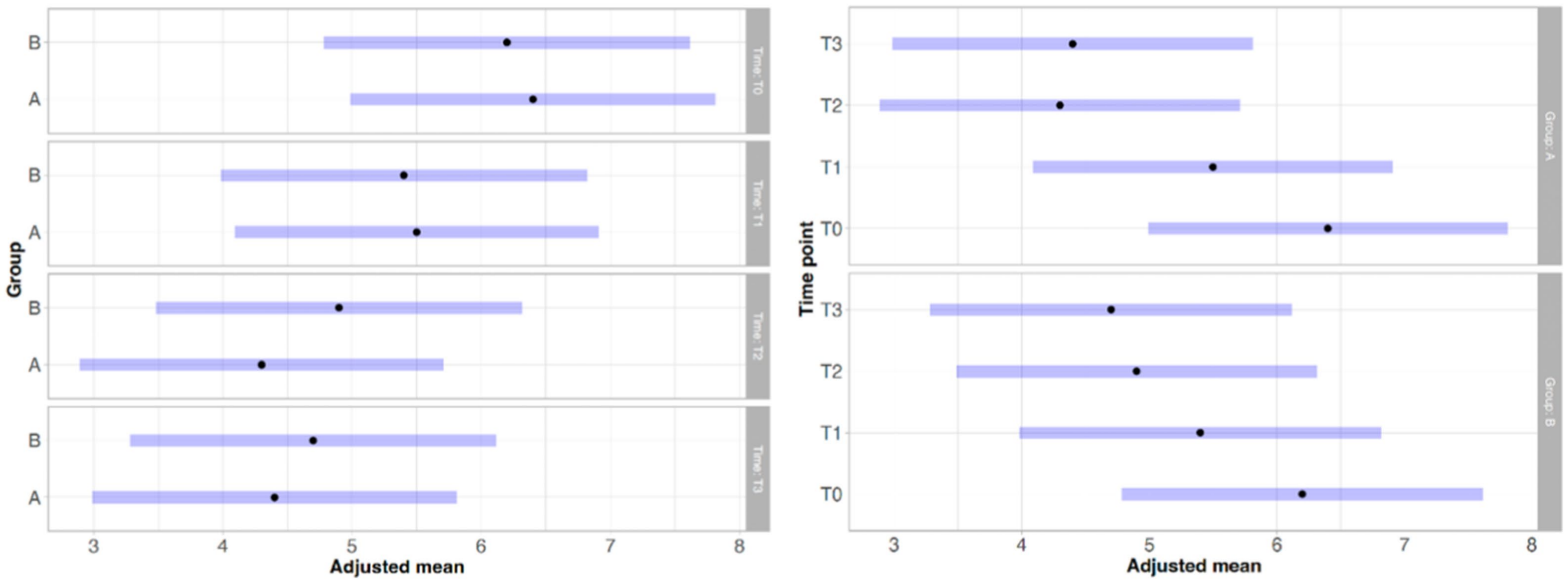
VAS pain scale analysis. No statistically significant differences were observed between Group A (Asudyn^®^) and Group B (Trocoxil^®^) (*p* > 0.05). Intragroup comparisons showed significant differences at T0–T2 (*p* < 0.001), T0–T3 (*p* < 0.001), and T1–T2 (*p* = 0.035) in Group A, and at T0–T2 (*p* = 0.019) and T0–T3 (*p* = 0.005) in Group B.

**Figure 5:**
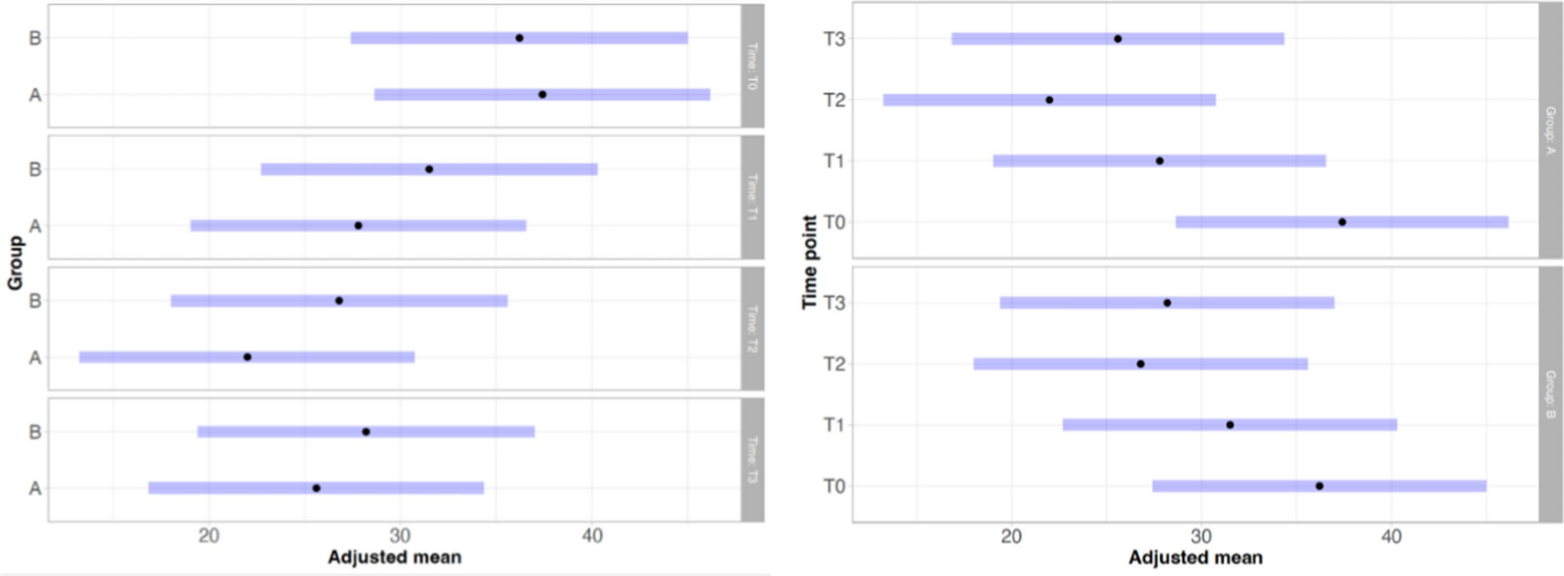
Statistical analysis of the CBPI Pain Interference Index (PII). No significant differences were found between Group A (Asudyn^®^) and Group B (Trocoxil^®^) (*p* > 0.05). Within-group analysis showed significant differences at T0–T2 (*p* < 0.001) and T0–T3 (*p* < 0.001) in Group A, and at T0–T2 (*p* = 0.011) and T0–T3 (*p* = 0.039) in Group B.

**Figure 6:**
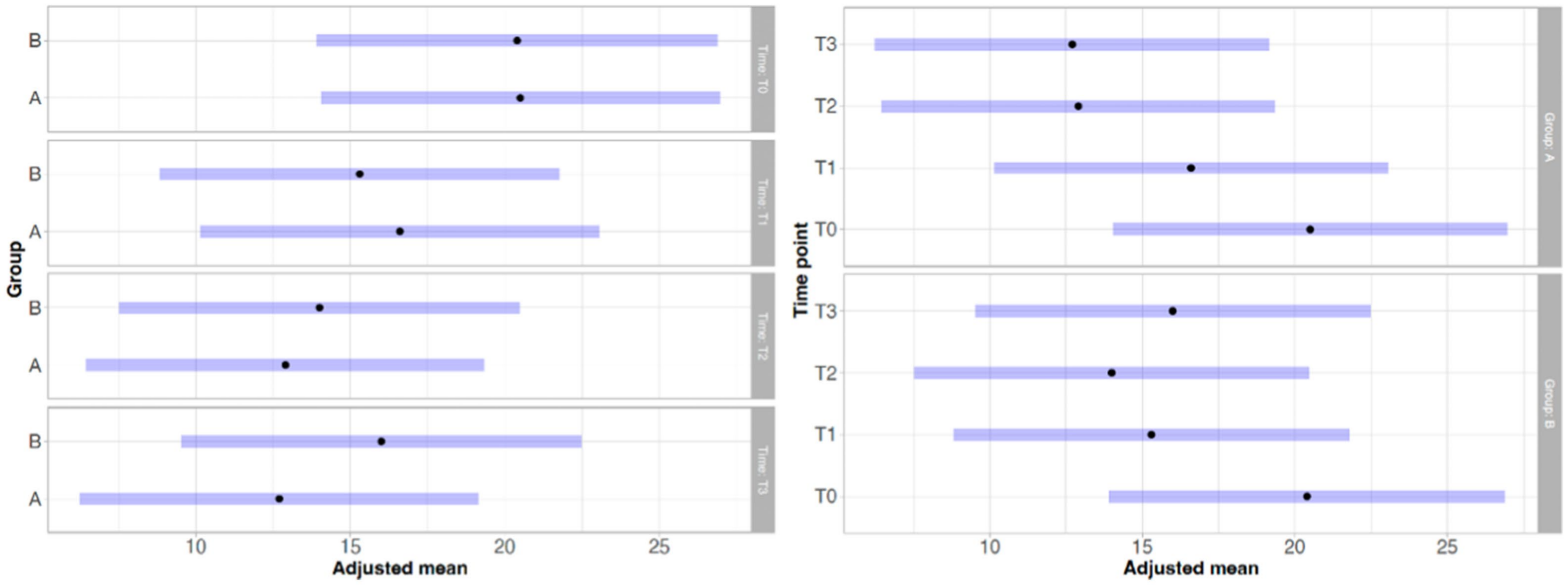
Statistical analysis of the CBPI pain interference index (PSI). No significant differences were found between Group A (Asudyn^®^) and Group B (Trocoxil^®^) (*p* > 0.05). Within-group analysis showed significant differences at T0–T2 (*p* < 0.001) and T0–T3 (*p* < 0.001) in Group A, and at T0–T1 (*p* = 0.025) and T0–T2 (*p* = 0.003) in Group B.

With regard to lameness assessment, both groups started from comparable baseline values at T0, with Group A showing a mean score of 1.83 and a standard deviation (SD) of 0.93, while Group B had a mean score of 1.80 and a SD of 1.13. As illustrated in Figure 1, the intra-group comparison across the different time points revealed a progressive improvement in lameness from T0 to T2 in both groups. However, while Group A continued to show improvement between T2 and T3, Group B exhibited a slight worsening of lameness at T3 compared to T2. Although both groups showed clinical improvement from T0 to T3, as depicted in Figure 1, statistical analysis did not reveal any significant differences either within each group over time or between the two groups at any given time point. Similarly, the results of the Gait Analysis indicated improvements in both TPI% and GLS% over the course of the study (T0–T3) in both groups. Specifically, Group A exhibited a steady and progressive improvement in TPI% (mean at T0 = 20.05; SD = 7.45) from T0 to T2, followed by a slight decline at T3 compared to T2 (Figure 2). In contrast, GLS% (mean at T0 = 80.51; SD = 25.85) progressively improved from T0 to T2 and then plateaued between T2 and T3 (Figure 3). In Group B, TPI% (mean at T0 = 20.17; SD = 8.41) demonstrated a slight and gradual improvement from T0 to T2, but showed a minor decline at T3 relative to T2 (Figure 2). The GLS% parameter (mean at T0 = 79.07; SD = 29.97) followed a similar trend, improving progressively from T0 to T2 and slightly worsening at T3 compared to T2 (Figure 3). No statistically significant differences were observed either between the groups or across the time points within each group for both TPI% and GLS% parameters, despite the clinical improvements recorded in both groups. Therefore, the results obtained from Gait Analysis are consistent with the findings of the clinical assessment of lameness severity. A general trend of improvement was observed in both study groups regarding pain-related parameters, as assessed by the Visual Analog Scale (VAS) and the Canine Brief Pain Inventory (CBPI), when comparing the initial evaluation to subsequent time points. Specifically, in Group A, the VAS score (mean at T0 = 5.90; SD = 2.60) showed a progressive improvement from T0 to T2, followed by a slight deterioration at T3 compared to T2. In contrast, Group B exhibited a steady and continuous improvement in the VAS score (mean at T0 = 5.88; SD = 2.94) throughout the study period from T0 to T3 (Figure 4). No statistically significant differences were found between the two groups at any individual time point. However, within-group analysis revealed statistically significant changes over time for both parameters. In particular, in the group treated with Asudyn^®^, significant differences were observed between T0 and T2 (*p* < 0.001), between T1 and T2 (*p* = 0.035), and between T0 and T3 (*p* < 0.001). In the Trocoxil^®^ group, significant improvements were found between T0 and T2 (*p* = 0.019) and between T0 and T3 (*p* = 0.005). Notably, the most pronounced statistical differences were recorded in the Asudyn^®^ group, especially for the T0–T2 and T0–T3 comparisons, with *p*-values < 0.001, indicating strong statistical significance relative to the standard threshold (*p* < 0.05) (Figure 4). Regarding the CBPI parameters, both the Pain Interference Index (PII) and the Pain Severity Index (PSI) demonstrated an overall trend of improvement in both study groups. In Group A, the PII (mean at T0 = 32.25; SD = 19.38) improved progressively from T0 to T2, followed by a decline at T3 compared to T2. A similar pattern was observed in Group B, where the PII (mean at T0 = 31.30; SD = 19.36) also improved until T2 and then worsened at T3 (Figure 5). The PS in Group A (mean at T0 = 19.91; SD = 11.70) showed a steady improvement from T0 to T2, with subsequent stabilization between T2 and T3 (Figure 6). In Group B, the PSI (mean at T0 = 17.00; SD = 13.68) followed a similar trend, improving from T0 to T2, followed by a deterioration from T2 to T3 (Figure 6). Statistically significant differences in PII were observed between T0 and T2, and between T0 and T3, in both groups. However, the Asudyn^®^ group demonstrated more pronounced significance, with *p* < 0.001 for T0–T2 and *p* = 0.001 for T0–T3 comparisons (Figure 5). As for the PSI, in the Asudyn^®^ group, comparisons between T0 and T2, as well as T0 and T3, yielded *p*-values < 0.001. In the Trocoxil^®^ group, statistically significant differences were noted between T0 and T1 (*p* = 0.025) and between T0 and T2 (*p* < 0.003) (Figure 6). No statistically significant differences were detected for the ROM (Group A at T0: mean flexion = 136.16, extension = 57.51; SD flexion = 44.38, extension = 14.02. Group B at T0: mean flexion = 138.9, extension = 46.6; SD flexion = 50.69, extension = 20.97), CRF (Group A mean T0 = 25.91; SD = 15.3. Group B mean T0 = 18.6; SD = 9.07), BCS (Group A mean T0 = 5.8; SD = 1.84. Group B mean T0 = 5.4; SD = 1.81) parameters or in the radiographic assessment (Group A mean at T0 = 2.91; SD = 1.37; Group B mean T0 = 1.9; SD = 1.37) either between the two study groups or across the time points within each group. However, no progression of OA was detected using the Kellgren-Lawrence grading scale at evaluation times T1 and T2 compared to T0. Similarly, no modification of CRF was observed during the evaluation periods.

### 3.3 Synovial score

**Figure 7:**
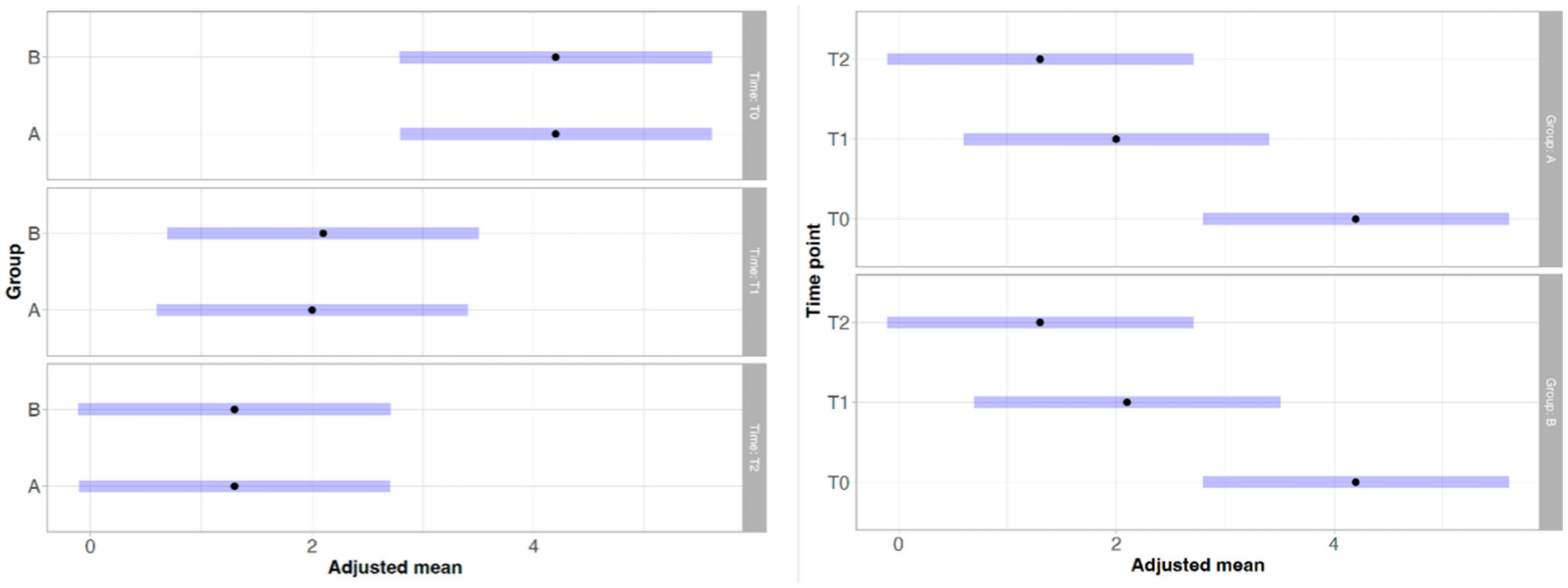
Statistical analysis of the synovial score (SS). No significant differences were observed between Group A (Asudyn^®^) and Group B (Trocoxil^®^) (*p* > 0.05). Intragroup comparisons revealed significant differences at T0–T1 (*p* = 0.007) and T0–T2 (*p* < 0.001) in Group A, and at T0–T1 (*p* = 0.010) and T0–T2 (*p* < 0.001) in Group B.

In Group A, the Synovial Score (SS) (mean at T0 = 3.66; SD = 2.53) exhibited a consistent improvement from T0 to T2. The most substantial reduction was observed between T0 and T1, followed by a less pronounced—though still evident—improvement between T1 and T2. A similar trend was observed in Group B, where the baseline SS (mean at T0 = 4.10; SD = 3.21) also improved progressively from T0 to T2. As with Group A, the most notable improvement occurred between T0 and T1, with a more moderate change from T1 to T2 (Figure 7). Although no statistically significant differences were found between the two groups at any time point, intra-group comparisons revealed significant changes over time in both groups. Specifically, in the Asudyn^®^ group, a statistically significant difference was observed between T0 and T1 (*p* = 0.007), and a highly significant difference was found between T0 and T2 (*p* < 0.001). Similarly, in the Trocoxil^®^ group, significant differences were recorded between T0 and T1 (*p* = 0.01) and between T0 and T2 (*p* < 0.001) (Figure 7).

## 4 Discussion

Recent years have witnessed a growing interest in exploring natural compounds, particularly phytoconstituents, renowned for their anti-inflammatory and joint-protective properties. The reason for this interest is likely to be found in the desire to limit or eliminate the adverse effects of NSAIDs. Although more selective NSAIDs have been developed over the years, the side effects have not been completely eliminated (62). Some interesting reviews on natural compounds therapy for osteoarthritis, both in humans and dogs, have identified several methodological issues in clinical trials: the limited number of rigorous randomized controlled trials and patient populations, the lack of objective outcome measures, the uncommon use of the concept of “effect size,” the risk of conflicts of interest, and the lack of standardization of dosages and treatment durations (63). Results of our research showed a trend of improvement in all the study times in both groups, without statistically significant differences between the two groups. Specifically, the pain and synovial fluid parameters showed a statistically significant reduction in both groups at the different study time points, with this significance being greater in the Asudyn group. The reason for these analgesic and anti- inflammatory effects of the product is likely attributable to the nature of the active ingredients contained in the complementary feed under study. The anti-inflammatory and analgesic effects of the individual components of the nutraceutical have, in fact, been widely documented and elucidated in the literature. Notably, the beneficial effects of essential polyunsaturated fatty acids (PUFAs) have been known for decades due to their unique properties in modulating the expression and activity of inflammatory biomarkers that cause articular cartilage degradation and in significantly reducing the pain associated with osteoarthritis, improving clinical symptoms in affected canine patients (64–66). Many research proved that essential polyunsaturated fatty acids (PUFAs) possess anti-inflammatory properties and for this reason they are considered an alternative to NSAIDs for the treatment of osteoarthritis (67, 68). *In vitro* studies have demonstrated that curcumin exerts both anti-inflammatory and antioxidant effects capable of slowing the degradation of the joint environment, thereby stabilizing the progression of chroni conditions such as OA (69). Specifically, it has been shown to suppress the activation of IκB (inhibitor of nuclear factor kappa-light-chain-enhancer of activated B cells, NF-κB) and the phosphorylation of Akt. Consequently, it prevents the translocation of NF-κB to the nucleus, thereby inhibiting the inflammatory response in chondrocytes. Furthermore, curcumin prevents the downregulation of mitochondrial Bcl-2 and Bcl-XL, preserving mitochondrial membrane potential and blocking chondrocyte apoptosis (70, 71). Thus, compared to other molecules, turmeric and curcuminoids exert their anti-inflammatory action not through the selective modulation of COX-1 activity (72), but through the modulation of nuclear factor kappa-light chain enhancer of activated B cells (NF-KB) signaling, the action in reducing pro-inflammatory cytokines such as prostaglandins, interleukins and leukotoxins, and finally through the activities operated on pro-inflammatory enzymes with a predilection for phospholipase α-2, cyclooxygenase-2 (COX-2) and 5-lipoxygenase (5-LOX) (25, 73–76). The beneficial effect of curcuminoids on modulating the inflammatory response at the joint level in dogs has been demonstrated through objective assessments, which also made use of a force platform, which showed an increase in peak vertical strength of the affected limb in subjects treated with curcuminoids (28, 77–79). The anti-inflammatory and analgesic properties of *B. serrata* extracts have been demonstrated both *in vitro* studies (63, 80, 81) and *in vivo* (63, 82, 83) It exerts a protective effect on articular cartilage by reducing glycosaminoglycan degradation. Boswellic acids (BAs) are extracted from the oleo-gum resin of *Boswellia serrata*. Among the pentacyclic triterpene acids identified, four have been shown to exert anti-inflammatory activity in joint tissues: β-boswellic acid, 3-acetyl-β-boswellic acid, 11-keto-β-boswellic acid, and 3-acetyl-11-keto-β-boswellic acid. BAs act by selectively inhibiting 5-lipoxygenase in a non-redox, non-competitive manner (84, 85). These compounds also inhibit the synthesis of leukotrienes and prostaglandins—key mediators of inflammation—and reduce the production of interleukins and TNF-α, molecules directly involved in cartilage tissue destruction (85). ASUs exert chondroprotective effects by modulating both catabolic and anabolic pathways involved in the development and progression of joint lesions. They have also been shown to stimulate collagen synthesis in chondrocytes, potentially contributing to the prevention of early cartilage degradation typically seen in OA (86–88). Notably, one study assessed the therapeutic potential of ASU in a canine model of experimentally induced OA. The results revealed a significant reduction in both macroscopic and microscopic cartilage lesion severity (22). The results of our study differ from those reported by Boileau et al., as we did not observe any radiographically visible improvement in the joint components. However, to properly assess cartilage damage and its progression post-therapy, the use of diagnostic tools such as Magnetic Resonance Imaging (MRI) would be indicated. MRI would allow for the visualization of direct signs of articular cartilage healing, unlike the indirect findings that can be evaluated using radiographic examination. Many animal models and *in vitro* trials possibly explained the mechanisms of efficacy from the willow bark extract. For instance, willow bark extract inhibits proinflammatory cytokines, such as tumor necrosis factor α (TNFα), cyclooxygenase-2 (COX-2), and the nuclear translocation of the transcription factor in proinflammatory activated monocytes, resulting in its anti-inflammatory effect (88). Willow bark extract is now widely used for conditions associated with inflammation (55). For arthritis per se, the efficacy of willow bark from various studies is diverse. Three studies with RCT showed that both willow bark extract alone (56) and compound drugs (57, 58) had an analgesic effect versus placebo. In contrast, three studies with RCT showed that willow bark extract yielded no significant benefit (59, 60). According to Biegert et al., although salicin derivatives in the willow bark were metabolized *in vivo* to salicylic acid, serum salicylate concentration was too low to reach clinical effects (59). In addition, the inhibition mechanism of the COX-2- mediated release of prostaglandin E2 was confirmed *in vitro*, but still short of proof of *in vivo* trials (61). The greatest biological activity of of Harpagophytum (devil’s claw) was attributed to the iridoid compounds which are commonly attributed anti-nociceptive, analgesic, antimicrobial, neuroprotective and anti-inflammatory effects through the inhibition of COX-2 and the expression of the specific mRNA for the inducible nitric oxide synthase (iNOS) gene, thereby reducing the production of prostaglandin E2 and nitric oxide, thus modulating the progression of the inflammatory processes involved in the osteoarthritic process (89–93). A study in canines evaluated the efficacy of Harpagophytum in improving the clinical manifestations of osteoarthritis, demonstrating that those treated with this nutraceutical showed a significantly higher improvement in peak vertical strength, measured with a force plate, compared to untreated dogs (94). The phytocomplex Asudyn^®^ was formulated based on the hypothesis that its individual components exert synergistic effects—a hypothesis supported by extensive evidence in both human and veterinary medical literature (44–48). Indeed, some studies have investigated the combined effect of multiple natural active ingredients in the treatment of osteoarthritis (95). Martello et al. (96) documented a progressive decrease in pain levels in 13 dogs treated for 60 days with a nutraceutical supplement containing *Boswellia serrata*, *Curcuma longa*, glucosamine, chondroitin sulfate, and type II collagen. Similar with our study, all dogs presented with spontaneous osteoarthritis and exhibited progressive improvements in both pain and lameness scores relative to baseline. Our results are also in agreement with those of Kimmatkar et al. (97), in which 20 client-owned dogs affected by spontaneous OA were orally treated with a supplement containing *Curcuma longa* and boswellic acid for 60 days. The findings of this study demonstrated a significant reduction in pain perception at each evaluation point, specifically after 45, 60, and 90 days from baseline. Pain reduction is a very frequently reported effect in clinical studies regarding the treatment of OA with natural components, both in human and veterinary medicine, and is consistent with what was found in our study (76, 97–100). The present study was conducted with the second specific aim of providing rigorous evidence regarding the therapeutic potential of the study supplement to alleviate the clinical signs of canine OA and to identify the occurrence of any adverse effects. To address the limitation of the absence of an objective and standardized system for lameness classification (101, 102), the GAITFourDog^®^ pressure platform system was employed to objectively assess weight distribution across the paw surface, thereby enabling quantitative gait analysis (37, 60, 94). This method facilitated the evaluation of limb placement, loading, unloading, and mobility restrictions through precise and reproducible metrics, unlike subjective visual assessments which are prone to observer bias and lack clinical objectivity (103, 104). Although both GLS% and TPI% values improved during the study relative to baseline (T0), these changes did not achieve statistical significance, yet they corroborate the clinical findings derived from the modified Numerical Rating Scale (NRS), thereby supporting the reliability of both assessment methods. Cytological examination of the synovial fluid conducted at 0, 30, and 60 days and performed by a highly experienced pathologist provided information regarding the degree of joint inflammation, allowing for objective support of the clinical data. Cytological analysis of synovial fluid provided additional insight into joint health and potential prognostic markers (105, 106). A statistically significant reduction in joint inflammation was observed in both groups compared to baseline. In particular, synovial fluid quality markedly improved after 60 days of treatment, with *p*-values < 0.001, and these changes were statistically comparable between the nutraceutical and NSAID-treated groups. Another peculiarity of our study was the choice of an NSAID for the control group, rather than a placebo like most studies with a control group. Mavacoxib (Trocoxil^®^) was chosen as the control therapy for this study due to its distinctive pharmacokinetic profile, characterized by low clearance and a high apparent volume of distribution. This allows for effective management of OA-related pain at a significantly lower therapeutic dose than other NSAIDs such as carprofen, celecoxib, or deracoxib (107–109). Its pharmacokinetics confers a prolonged half-life, which supports a unique dosing regimen: initial doses 2 weeks apart, followed by maintenance doses every 4 weeks (51, 108). As reported by Payne-Johnson et al. (51), this extended interval offers advantages over daily dosing, especially in patients with poor compliance or increased susceptibility to drug-related adverse effects. In the group treated with Trocoxil^®^ (mavacoxib), a COX-2-selective NSAID, significant improvements in both pain and lameness were observed during the study period compared to baseline. Interestingly, clinical outcomes were comparable to those observed in the Asudyn^®^ group, suggesting that the natural formulation may offer similar clinical benefits to Trocoxil^®^, recognized in recent literature as a highly effective NSAID for the management of OA, while avoiding the risk of adverse effects typically associated with long-term NSAID use (51, 96, 110–113). The primary limitations of this study include the absence of long-term follow-up (beyond 1 year post-treatment) and a relatively small sample size. Another limitation relates to the heterogeneity of the study population in terms of breed, age, and baseline osteoarthritis (OA) severity. Further studies are warranted to analyze larger patient cohorts with more homogenous osteoarthritic conditions. Furthermore, the inclusion of a placebo control group could confirm and validate the efficacy of the nutraceutical blend demonstrated in this study and mitigate a possible bias effect. A general limitation concerns the use of natural compounds. These are extracted from herbal products or other environmental sources, and their active compound content is influenced by extraction and purification strategies, local plant growth conditions (e.g., soil composition), and the frequently used oral administration route, which is associated with uneven uptake and often inconsistent bioavailability (44). Therefore, unfortunately, maintaining the same composition of Asudyn but changing the source of the active ingredients or their concentration could lead to different results. It would therefore be useful to study these issues thoroughly and find qualified centers that produce these components. A possible answer to the need to improve the bioavailability of these substances comes from very recent studies on the integration of nanotechnology with phytoconstituents. This interaction emerges as a promising strategy, addressing the limitations of traditional arthritis treatments. Nanocarriers such as liposomes and nanoparticles provide a platform for targeted drug delivery, improving the bioavailability of phytoconstituents. This circumvents the inherent problems of poor solubility, stability, and bioavailability. Nanocarriers offer solutions that improve pharmacokinetics and allow for sustained release, thus increasing overall therapeutic efficacy (62). Another limitation of the study is the lack of evaluation of the blood and synovial concentration of the individual components during the study period. This limitation prevents us from definitively asserting that all components of the nutraceutical contributed to the therapeutic effect, or if the benefits resulted from only a few or even a single component, especially considering that the literature has already extensively demonstrated the therapeutic capabilities of the individual components (44–48, 84, 85, 88, 90–94). However, the concentrations of the single components within Asudyn appear to be relatively low to justify such a satisfactory therapeutic effect (69, 77–79, 82, 83). This consideration supports the initial hypothesis that the various components exert a synergistic effect, thus allowing them to produce the therapeutic effects shown in the results even at lower concentrations (95–97). It would therefore be interesting to perform a further *in vivo* study to evaluate the concentrations of the single components reached in the blood and/or at the joint level during the treatment period in order to substantiate this hypothesis. Furthermore considering that the results of this study have demonstrated significant efficacy of the pharmaceutical blend, in some aspects comparable to the use of anti-inflammatories as discussed earlier, it is imperative to conduct a further study incorporating a placebo group as a control to validate the results obtained and mitigate any potential biases.

## 5 Conclusion

The results of our study demonstrated that Asudyn^®^ was effective in reducing pain, decreasing lameness severity, and improving the qualitative characteristics of synovial fluid in joints affected by osteoarthritis (OA). Specifically, regarding the reduction of pain and lameness, these effects were comparable to those observed in subjects treated with mavacoxib. However, regarding the improvement of synovial fluid quality, Asudyn^®^ demonstrated greater effects. The therapeutic benefit of Asudyn^®^ could be attributed to its specific formulation and the carefully balanced concentrations of its active ingredients. In conclusion, Asudyn^®^ demonstrated comparable efficacy to mavacoxib in the treatment of OA, with the added advantage of not presenting the adverse effects commonly associated with the use of NSAIDs. Therefore, it is believed that it can be used effectively part of a multimodal treatment regimen in the treatment of osteoarthritis, ensuring efficacy and the reduction of NSAIDs adverse effects.

## Data Availability

The raw data supporting the conclusions of this article will be made available by the authors, without undue reservation.
